# Heat Stress: A Serious Disruptor of the Reproductive Physiology of Dairy Cows

**DOI:** 10.3390/ani13111846

**Published:** 2023-06-01

**Authors:** Eleni Dovolou, Themistoklis Giannoulis, Ioannis Nanas, Georgios S. Amiridis

**Affiliations:** 1 Laboratory of Reproduction, Faculty of Animal Science, University of Thessaly, 41223 Larissa, Greece; entovolou@uth.gr; 2 Department of Obstetrics & Reproduction, Faculty of Veterinary Science, University of Thessaly, 43100 Karditsa, Greece; gnsnanas@gmail.com; 3 Laboratory of Genetics, Faculty of Animal Science, University of Thessaly, 41223 Larissa, Greece; thgianno@uth.gr

**Keywords:** heat stress, endocrine response, gene expression, fertility, dairy cow

## Abstract

**Simple Summary:**

Global warming has caused a significant extension of the duration and the mean temperatures of summers; this is a serious stressor for dairy cows that are particularly sensitive to the high temperatures. Under these conditions, the productivity and the fertility of the animals are seriously compromised leading to considerable financial losses to the farmers. A deep understanding of the prevailing mechanisms of the cow to withstand this serious stressor, is prerequisite for planning and implementation of heat stress mitigation programs. To this end, in this review we analyze the main hormonal and molecular alterations that take place during heat stress, and which directly or indirectly affect cow’s fertility.

**Abstract:**

Global warming is a significant threat to the sustainability and profitability of the dairy sector, not only in tropical or subtropical regions but also in temperate zones where extreme summer temperatures have become a new and challenging reality. Prolonged exposure of dairy cows to high temperatures compromises animal welfare, increases morbidity, and suppresses fertility, resulting in devastating economic losses for farmers. To counteract the deleterious effects of heat stress, cattl e employ various adaptive thermoregulatory mechanisms including molecular, endocrine, physiological, and behavioral responses. These adaptations involve the immediate secretion of heat shock proteins and cortisol, followed by a complex network of disrupted secretion of metabolic and reproductive hormones such as prolactin, ghrelin, ovarian steroid, and pituitary gonadotrophins. While the strategic heat stress mitigation measures can restore milk production through modifications of the microclimate and nutritional interventions, the summer fertility records remain at low levels compared to those of the thermoneutral periods of the year. This is because sustainment of high fertility is a multifaceted process that requires appropriate energy balance, undisrupted mode of various hormones secretion to sustain the maturation and fertilizing competence of the oocyte, the normal development of the early embryo and unhampered maternal—embryo crosstalk. In this review, we summarize the major molecular and endocrine responses to elevated temperatures in dairy cows, as well as the impacts on maturing oocytes and early embryos, and discuss the consequences that heat stress brings about in dairy cattle fertility.

## 1. Introduction

Global warming is defined as the long-lasting and steady increase in the global annual temperature. Since the early 1980s, the global temperature has been increasing by approximately 0.18 °C per decade, leading to a projection that by 2100, the Earth’s temperature could rise by 2.1 °C to 3.9 °C [1,2]. This alarming situation has triggered a cascade of abnormal climatic phenomena, including ice melting, rising sea levels, and severe weather anomalies such as prolonged heat waves, extended droughts, and floods. Similar abnormal climatic conditions have been observed during previous centuries, for example the medieval warm period (MWP), where annual mean temperatures were similar to those of the end of the 20th century. However, natural factors can account for the MWP temperature changes, whereas only anthropogenic interventions can explain the current anomalous global warming [3].

**Figure 1:**
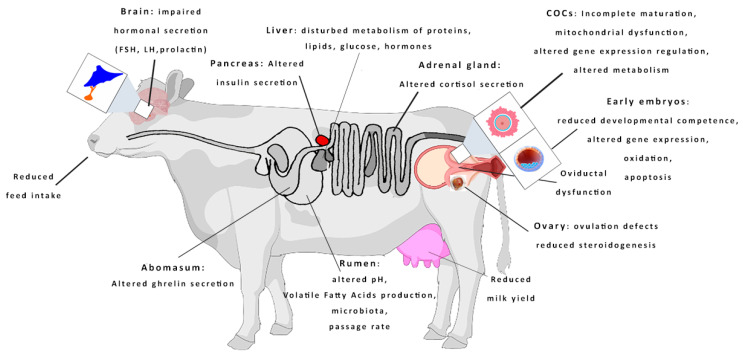
Schematic representation of important sites of HS action on dairy cows that directly or in directly affect their reproductive performance.

The extreme summer temperatures and the annual temperature rise have been recognized as one of the gravest threats to global agriculture. The dairy sector is particularly sensitive to the climate change, as heat stress (HS) leads to increased incidences of infectious and metabolic diseases, reduced milk production, and waned fertility of lactating cows over time. When dairy cows are exposed to temperatures beyond the upper threshold of their thermoneutral zone (16 to 25 °C) [4], they become stressed. High relative humidity, in combination with increased temperatures, can exacerbate the degree of HS of cows. To accurately evaluate the climatic conditions conducive to cow comfort, the temperature humidify index (THI) has been adopted. According to the THI, cows are in the comfort zone at THI < 68, mildly stressed at THI between 69–78, moderately stressed at THI 79–89, and severely stressed at THI > 90 [5]. However, it should be noted that the thresholds set to define thermal comfort can vary depending on different factors (breed, production level, skin color, etc). Almost all European breeds of dairy cows are extremely vulnerable to HS due to several factors. These breeds have been traditionally raised in colder climates and lack genetic adaptations to cope with HS. Additionally, their extremely high metabolic rate associated with milk synthesis significantly increases internal heat production [6]. To withstand prolonged exposure to HS, cows activate a series of thermoregulatory mechanisms, including behavioral, physiological, endocrine, and molecular responses [7], as shown in Figure 1.

Behavioral changes are the initial response to HS. In this category, shade-seeking [8,9] and lying-down behaviors are affected. As the severity of the HS increases, dairy cows spend more time standing instead of lying down. This behavior may be related to the effort of the animals to dissipate heat through convection and evaporation [9]. This altered behavior degrades welfare and predisposes cows to health, production, and fertility problems. Under HS, lactating cows exhibit increased water consumption and decreased dry matter intake. In the summer, water intake can increase two- to four-fold due to water loss through evaporation, sweating, panting, and urination, resulting in blood hyperosmolarity [5,10]. On the other hand, dry matter intake is significantly decreased, leading to negative energy balance, which is a major contributor to reduced milk production and compromised fertility [11,12,13]. While the reduction in dry matter intake during HS is well-established, it has been shown that approximately 35–50% of the reduction of milk production can be attributed in the energy deficit, while feed intake-independent, postabsorptive shifts in glucose and fat metabolism, sparing, and homeostasis contribute to the remaining milk production losses [14,15].

In recent decades, the preservation of high milk production during the hot months has made the implementation of cooling management systems an inextricable need. Indeed, the installation of sophisticated automated cooling systems has allowed for maintining summer milk production close to 98% of winter levels. However, strategic cooling cannot restore summer fertility, which exhibits a 30% reduction compared the winter fertility rates [6]. This reduced summer fertility poses a significant economic threat to the profitability and sustainability of farms. The summer subfertility is the outcome of the cumulative effects of stressors on all tissues and systems that directly or indirectly regulate the functionality of the reproductive system. These effects disrupt the balance of reproductive hormones, impair oocyte quality, and hinder proper embryo development. As a result, weak estrus expression, low conception rates, and increased embryo mortality are synergistically observed [16,17,18,19,20]. The aim of this review is to discuss the reproductive endocrinologic implications of heat-stressed dairy cows and to focus on the effects of high temperatures on oocyte fertilizing capacity and early embryo development.

## 2. Heat Shock Response: The First Line of Molecular Defense Mechanisms against Heat Stress

In all mammals, the first molecular reaction against thermal stress is the synthesis of heat shock proteins (HSPs). These proteins exert a protective role in cells by safeguarding cellular integrity, immune response, apoptosis, and contributing to post-translational folding and transportation of proteins through cellular membranes [21,22,23,24,25,26].

It was initially believed that HSPs were exclusively associated with the response to thermal stress, but it is now clear that almost all stressors induce HSP expression [27]. HSPs are classified according to their molecular weight into small (MW < 40 kDa) and large (MW > 40 kDa) categories. Under HS conditions, the mRNA expression of both small and large HSPs is significantly upregulated in many cell types [28,29,30,31]. The transcription of HSPs is primarily regulated by heat shock factors (HSFs), which are encoded by the HSF gene family. These genes are present in mammals and have been implicated in the HS response [32]. Recently, specific genotypes of the *HSF1* gene have been identified as possible causal variants for thermotolerance in dairy cows [33].

Among the various types of HSPs, HSP70 has garnered particular attention from the research community. At thermoneutral conditions, it is present in all cellular compartments, while during and after HS, it is predominantly present in the nucleus [34]. It appears that HSPs can serve as indicators of HS tolerance in cattle. Increased mRNA expression and HSP70 synthesis by peripheral blood mononuclear cells (PBMC) has been considered as a strong indication for thermotolerance and conserved cellular viability, which are attributed to specific variants of HSP70 [35].

In Zebu cattle, which are evolutionary more heat resilient than continental breeds, Sailo et al. [36] found that specific genotypes of *HSP90* genes are associated with thermotolerance, resulting in unaffected body temperature and milk yield during HS.

Breed-specific responses to HS were also noted in our previous study, comparing the reproductive potential of heat-stressed oocytes from Holstein and Limousine cattle. The oocytes derived from Holstein exhibited a stronger response against HS, as evidenced by specific changes in gene expression and a higher suppression of in vitro blastocysts formation compared with Limousine oocytes. These changes may be attributed to direct or indirect selection of Holstein cattle, which are farmed in intensive systems [18]. Diaz et al. [37] described the transcriptomic changes of oocytes during HS using RNA sequencing and highlighted specific pathways and ontologies affected by high temperatures. One of the most interesting findings was the negative regulation of transcription resulting from the activation of the unfolded protein response (UPR) regulated by *HSF1*.

In a recent study [31], we examined the response to HS in two groups of Holstein cows characterized as phenotypically thermotolerant or thermosensitive based on their fertility under harsh summer environmental conditions. It was found that when the THI was >80, thermotolerant cows had significantly lower concentrations of HSP70 and cortisol compared to thermosensitive cows. This suggests that these phenotypically resilient individuals have a higher threshold for HS. In a follow-up study involving phenotypically sensitive and tolerant animals, a whole-genome sequencing approach was used to determine specific pathways associated with adaptive mechanisms of thermotolerance. These pathways encompassed systemic and cellular responses, including the expression of HSPs, unfolded protein response, immune system functionality, and regulation of insulin production pathways. These specific pathways revealed specific parts of the genetic basis of the phenotypical characteristics, and allowed for the annotation of specific SNPs (e.g., rs135169588, rs41255191, rs41964478, and rs136591078) mapped in five genes encoding HSPs (*DNAJC27*, *DNAJA4*, *DNAJC21*, *DNAJB1*, and *HSPA1A*). These SNPs could be used for genetic selection for thermotolerance [38].

Other genetics and genomics studies have highlighted the importance of identifying loci associated with thermotolerance, which can be applied to genetic/genomic selection programs to enhance the genetic component of thermotolerance in the herds. Cheruiyot et al. [33] conducted a GWAS analysis of 30,000 animals and found specific genes and pathways associated with thermotolerance. Among them, genes related to the neuronal system (*ITPR1*, *ITPR2*, and *GRIA4*) and neuroactive ligand–receptor interaction (*NPFFR2*, *CALCR*, and *GHR*) were revealed, offering new insights for the development of genetic selection approaches. In a recent study, Luo et al. [39] used a combination of GWAS and RNASeq techniques, leading to the discovery of important mechanisms related to heat stress response, including *PMAIP1*, *SBK1*, *TMEM33*, *GATB*, *CHORDC1*, *RTN4IP1*, and *BTBD7.* Otto et al. [40] used a total of 476 animals in a GWAS analysis to investigate heat stress response and identified four genes (*LIF*, *OSM*, *TXNRD2*, and *DGCR8*) involved in biological processes associated with heat stress, serving as putative markers for genetic selection in Holstein cattle. Other studies utilized single gene association approaches [41,42,43] and detected several genes associated with thermotolerance in cattle, such as *PRLH*, *SOD1*, *ATP1A1*, and *HSP70A1A*. Zeng et al. [44] reviewed several of these efforts and highlighted the main genes associated with thermotolerance identified through such approaches.

There is strong evidence implicating HSP70 in oocyte fertilization and early embryo development [45]. It is well-documented that during HS, HSP70 acts protectively by contributing to stabilization of the cytoskeleton and regulating immune response and cell cycle [26,46]. It is also believed that HSP70 acts as an anti-apoptotic agent by interrupting the mechanism of caspase 3 activity [47] and reducing the phosphorylation of elF-2a, which is an initiation factor for protein translation [46]. Collectively, it appears that HSP70 mediation confers cellular resilience against thermal insults by mediating these processes.

Cattle cumulus oocyte complexes (COCs) respond to HS by increasing the mRNA expression of HSPs during in vitro maturation when exposed to high temperatures [48]. Hence, HSPs, in addition to modulating apoptosis and oxidation pathways, can also serve as markers of cellular stress that affects oocyte competence [49]. Souza-Cacares et al. [50] studied the expression of HSPs in COCs collected from two tropical cattle breeds (Gir and Pantaneiro) using oocyte pick-up and found that the mRNA expression and the concentration of HSP70 and HSP90 varied between breeds. Their results suggest that higher production of HSP70 is positively associated with the intrinsic thermotolerance of the breed. Similarly, differences in mRNA expression of *HSP90AA1* were detected between Holstein and Limousine oocytes that were subjected to moderate heat stress conditions during in vitro maturation [18]. It is well-documented that exposing in vitro maturing bovine oocytes to 41 °C for 8, 12, or 24 h has deleterious effects on embryo production [51,52,53,54].

Before the fourth cell division, early embryos are extremely sensitive to HS. At this stage, the embryonic genome is activated [55,56] and the early embryo acquires a gradually increasing thermotolerance as its development progresses to the blastocyst stage [57,58,59]. During the early stages of embryonic development, increased transcription activity and protein synthesis is observed at the time when the embryonic genome becomes activate [60,61]. At this stage, several HSPs [62] and antioxidant enzymes [63] are overexpressed, strengthening the molecular “arsenal” of thermotolerance in the developing embryo. Nevertheless, it has been documented that transcriptional activity for HSPs and HSP70 synthesis is detectable even before embryonic genome activation [64,65]. It has been documented that early embryos express a rapid reaction to short-term moderate temperature increases, which is evidenced by the steadily increasing expression of mRNA for HSPA1A and HSP90AA1 after exposing early morulae to a 1.5 °C elevated temperature (40.0 °C instead of 38.5 °C) for 2 to 8 h [63].

## 3. Endocrine Response to Heat Stress

Heat stress has been long recognized as a threatening environmental condition for cattle fertility [66,67,68], and the underling neuroendocrinological imbalances [69,70] and alterations in the structure and functionality of follicles and enclosed oocytes have been meticulously investigated [71,72,73]. Despite the accumulated knowledge, there are still many aspects of fertility-regulating mechanisms under HS that remain elusive. In vitro studies provide a reliable model for studying the effects of HS on follicular components such as follicular fluid composition, functionality of the thecal, granulosa, and cumulus cells, as well as the enclosed oocyte. The undisturbed ovarian functionality is achieved through an endogenous thermoregulation system that maintains the ovarian temperature at lower levels than the rectal or the neighboring tissues temperatures. This is particularly important for the preovulatory follicle, where the internal temperature is 0.5 to 1.5 °C lower than that of the surrounding ovarian tissues [74,75,76]. Prolonged HS dysregulates the endogenous ovarian cooling system, exposing the oocyte to higher-than-normal temperatures, which impairs its fertilizing capacity. In vitro experiments have provided a large body of information on the effects of HS on the fertilizing capacity and the quality of in vitro-produced embryos. However, the experimental conditions used in these experiments do not necessarily mimic the in vivo environment.

### 3.1. Reproductive Endocrinology under Heat Stress

The ovarian function is regulated by an orchestrated interplay of hypothalamic gonadotrophin-releasing hormone (GnRH), anterior pituitary gonadotrophins (follicle-stimulating hormone—FSH and luteinizing hormone—LH), gonadal steroids (progesterone and estradiol), and follicular peptides (inhibin, activin, follistatin). However, it is now well established that several other hormones and metabolites, such as glucose, cortisol, growth hormone, prolactin, ghrelin, and kisspeptin, affect gonadal function, acting centrally (hypothalamus, pituitary gland) as well as in peripheral tissues (e.g., ovaries).

The deviated follicular development during HS should primarily be linked to perturbated hypothalamus–pituitary–gonadal (HPG) axis communication, resulting from a vicious cycle that includes impaired gonadotrophic support (either directly or mediated by the interference of other extraaxial stimuli) and suboptimal ovarian steroidogenesis. In many reports, the deviated HPG functionality has been examined independently of alterations occurring due to HS in the metabolic status of the animals. For example, during HS, the dry matter intake of the cows is decreased [77], leading to a negative energy balance. It is well established that GnRH neurons are sensitive to alterations of glucose and various metabolic signaling factors (insulin, leptin, IGF-1, melanocortin, kisspeptin) during periods of low energy balance availability [78,79], which commonly occur during the hot periods of the year. During the summer, concentrations of acylated ghrelin, which has a regulatory role in gonadotrophin secretion, are affected differently in lactating cows and pregnant heifers [80,81]. Kisspeptin, which is synthesized mainly in preoptic area and the arcuate nucleus of the hypothalamus, it is considered as an important regulator of GnRH and LH synthesis, but it also has a metabolic role as it is involved in the release of growth hormone [82,83]. It has been shown in many species that various stressors seriously affect kisspeptin and reproductive physiology. For example, feed withdrawal for 72 h caused downregulation of kisspeptin expression, suppressed LH secretion, and extended the duration of the estrous cycle in rats [84]. Similarly, hypoglycemia, a serious metabolic stress, was induced in rats through insulin infusion, causing profound inhibition of LH pulsatility, which was associated with the downregulated expression of Kiss1 and Kiss1r (receptor) mRNA [85]. In pigs, under high summer temperatures, the activity of kisspeptin neurons as well as the hypothalamic expression of kisspeptin were reduced compared to cooler periods of the year. The reduced kisspeptin expression was considered among the causative factors for weak gonadotrophin secretion and fewer large follicles during the summer [86]. Unfortunately, there is no available information on the effects of heat stress on kisspeptin expression in dairy cows.

### 3.2. LH

The effects on LH concentrations and secretion pattern (pulse frequency and amplitude) after exposure of cows to HS are discrepant, with some studies reporting no effects of HS on LH concentrations [87,88,89], others present higher LH concentrations [90], and others show decreased LH levels [70,91,92]. Reviewing the international literature, it appears that the latter scenario is the most plausible for the effects of HS on LH secretion. The inconsistency among studies on the LH concentrations and the mode of its secretion during or after HS can be attributed to different reasons. These include variations in study protocols, such as the use of different types of animals (lactating cows at different stage of lactation or heifers), short-term exposure to the stressor, field studies comparing hormone levels between thermoneutral and hot periods, and exposure of animals to different THI levels durations. Hence, the type and the intention of the stress, assessed by the cortisol levels, can modulate LH secretion. For example, when cows are exposed to castrated bulls, no changes in cortisol and LH secretion patterns are observed, but when the same animals are exposed to intact bulls, cortisol pulses frequency is reduced, and LH pulse amplitude and frequency are increased [93]. Additionally, during HS, phenotypically thermotolerant Holstein cows, which are characterized by lower cortisol and HSP70 concentrations, respond to exogenous GnRH with a higher preovulatory LH surge compared to that of thermosensitive herd mates [31]. Therefore, it is reasonable to assume that the type and the intensity of stress can regulate the mode of LH secretion.

Furthermore, when assessing the effects of HS on LH secretion, the estrogenic status of the animals should not be overlooked. In fact, reduced estradiol levels have been reported in many studies, which can explain the negative effects of HS on LH secretion [70,94,95]. In a detailed study, Gilad et al. [91] provided evidence that the LH pulse amplitude and the GnRH-induced preovulatory LH surge were attenuated in animals with low estradiol concentrations, but not in those with normal or high estradiol levels, highlighting the importance of estrogenic support in sustaining normal LH secretion. Abnormal estradiol secretion is likely among the primary reasons for summer subfertility in dairy cows [96], as it is responsible for short and weak estrus expression, increased incidence of silent ovulations [87], suboptimal follicular and oocyte maturation, and the development of corpora lutea with reduced secretory ability [97].

### 3.3. FSH

In an experiment involving controlled exposure of mature lactating cows to acute thermal stress during the winter and summer, Gilad et al. [91] provided evidence that HS manifested either as chronic summer HS or as abrupt exposure to high temperatures, affects gonadotrophin secretion during the follicular phase. GnRH induced FSH secretion during the early luteal phase was significantly reduced, but the suppression rate was much more profound in animals with reduced blood estradiol levels, which was possibly related to the suppressed steroidogenetic ability of the granulosa cells caused by previous exposure to HS. Similarly, no difference was detected in FSH secretion in young heifers after controlled exposure to HS [89]. However, it has been shown that in dairy cows exposed to elevated temperatures, the number of large follicles (>10 mm) in the first or ovulatory follicular wave, as well as the medium-sized follicles (6–9 mm) in the second follicular wave, dramatically increases [94,98]. This increase direclty indicates impaired dominance driven by elevated FSH concentrations and reduced inhibin levels throughout the estrous cycle [98]. In fact, the follicle-derived inhibin is a strong regulator of FSH secretion, exhibiting an inverse ratio with FSH concentrations [99,100]. Therefore, the subnormal inhibin concentration during HS must be attributed to an additional impaired function of granulosa cells.

### 3.4. Estradiol

Periovulatory estradiol 17β surge is suppressed in dairy cows that are exposed to controlled HS either during the mid and late luteal phase or throughout the entire estrous cycle [95]. Given that granulosa cells are the primary source of estradiol [101], the reduced estradiol concentrations observed during the hot periods of the year must be inevitably linked to the impaired steroidogenic potential of follicular granulosa cells. HS seriously affects granulosa cells, leading to increased oxidation, apoptosis, and reduced steroidogenetic capacity [102,103]. The HS-induced oxidation in granulosa cells is evidenced by the increased intracellular reactive oxygen species (ROS) accumulation [29,104]. It is interesting to note that increasing the culture temperature from 40 °C to 41 °C (both temperatures are beyond the thermoneutral zone) causes the ROS levels and apoptotic phenomena to decline, possibly due to upregulation of antioxidant genes such as superoxide dismutase 1 and 2 (*SOD1* and *SOD 2*) and glutathione-disulfide reductase (*GSR*). Furthermore, the upregulation of *HSP70* and *HSP90*, which protect cells against oxidative stress and activate self-protective mechanisms through the clearance of damaged proteins, is also observed [29]. In vitro culture of granulosa cells under elevated temperatures (41 °C) for 24 h attenuates the proliferation capacity of the cells. Along with higher ROS accumulation, it increases the expression of endoplasmic reticulum (ER) stress marker genes such as *GRP78* and *GRP94*, as well as the expression of apoptotic genes (*BAX* and *CASPASE-3*) compared to control cells cultured at 37 °C [105]. It has been shown that exposure of mammalian cells to high temperatures or to oxidation-promoting conditions can induce ER stress in mammalian cells. Under these conditions, ER homeostasis is disturbed, and abnormal (unfolder or misfolded) proteins accumulate in the ER, which activate the unfolded protein response (UPR) signaling pathway [106,107]. Collectively, under HS conditions, estradiol production by the granulosa cells is reduced, which can be attributed either to diminished expression of gonadotropin receptors [108] and/or the downregulation of many genes related to steroidogenesis, such as *CYP11A1* and *STAR* [29,102]. In addition, depressed estradiol synthesis by cumulus cells is evident in in vitro-cultured heat-stressed COCs, which is possibly due to abnormal regulation of FSH signal transduction [109,110].

### 3.5. Progesterone

While most studies agree that estradiol secretion is diminshed due to HS, this is not the case for progesterone, with the reports regarding the effects of HS on progesterone synthesis ranging from negative, neutral, or positive. When feed-restricted or normally-fed dairy heifers are exposed to moderate HS (THI = 84) for approximately 25 days (four days prior to expected estrus until the following ovulation), from day nine of the estrous cycle, progesterone concentrations are significantly lower in heat-stressed animals compared to controls or feed-restricted heifers that were kept under thermoneutral conditions [89]. After controlled and abrupt exposure to HS, Wilson et al. [95] reported that the duration of the estrous cycle is prolonged, possibly due to delayed luteolysis, while the mid-luteal secretory ability of the corpus luteum is not affected by the HS. In another study with a similar methodology, the progesterone levels during the second half of the cycle of the heat-stressed cows were higher than those of the non-heated controls [111]. Follicular wall samples obtained at thermoneutral periods of the year and subsequently cultured at 40 °C demonstrated increased progesterone synthesis and suppressed estradiol and androstenedione compared to non-heated controls [112]. In an early in vitro study, luteal cells collected during the winter showed increased viability compared to those collected in the summer. Additionally, granulosa cells collected during winter and in vitro cultured at 38 °C produced 30% more progesterone compared to those cultured at 40 °C [113]. The variability in findings on progesterone concentration after exposing the animals to HS is difficult to interpret. These discrepancies can be attributed to many factors, such as the synthesis rate by the CL, the clearance rate in the liver, which is affected by milk production, the synthesis in extra-ovarian tissues (e.g., adrenal gland), the duration and intensity of exposure to the stressor, the availability of cholesterol, which is the precursor for progesterone synthesis, etc. We provided evidence that during the 5th week of pregnancy, mean progesterone concentrations are lower during the hot season of the year compared to that of thermoneutral periods [114]. This reduced progesterone secretion under HS conditions persists until at least until day 100 of the pregnancy. At this stage, we used Doppler ultrasonography to assess the echotexture and the blood supply in the corpus luteum. It was revealed that, under HS, the mean gray-scale intensity of corpus luteum was lower and the color flow was higher than in the winter. As there is a strong association between echogenicity, blood flow, and the secretion ability of the corpus luteum, [115,116] it is very likely that in the summer, the luteal tissue is equally or more vascular compared to that of the winter. However, the luteal cell density is reduced, explaining the lower progesterone levels [114]. Given this evidence, Wolfenson et al. [117] inferred that the luteal insufficiency during HS is the result of inadequate luteinization of the preovulatory follicle. Accumulated knowledge suggests that the short-term HS does not interferes with luteal progesterone synthesis, while long-term exposure to HS (such as the naturally occurring during the summer) seriously impairs the secreting ability of the corpus luteum [6,117], leading to devasting effects on follicular development, oocyte maturation, and early embryo survival [20,118,119].

### 3.6. Cortisol

The pathway of HS-induced cortisol secretion involves the initial stimulation of the para-ventricular neurons to secrete corticotropin-releasing hormone (CRH), which acts on the adenohypophysial corticotrophs, stimulating ACTH secretion and inducing cortisol synthesis by the adrenal cortex. In an early work, it was shown that within 20 min, dairy cows respond to HS by increasing cortisol secretion. If the stimulus persists, cortisol levels steadily increase and plateau at 2 to 4 h. However, under continuous (7 weeks) exposure to high temperatures, an acclimation pathway is activated, and the levels drop to suprabasal levels [120]. The HS-induced cortisol secretion has been documented in many studies. When dairy cows and calves have access to shade, they exhibit lower blood cortisol concentrations compared to cows [90] and calves [111] kept under direct solar radiation. After exposing crossbred cattle to different thermal insults, it has been shown that the rise in cortisol concentrations is temperature-dependent [121]. Similarly, milk cortisol concentrations in Holstein cows reared at THI = 80.5 are significantly higher than in those kept in the thermoneutral zone [122]. Continuous chronic exposure to high THI depresses cortisol concentration in Holstein heifers, possibly due to a complex acclimation pathway involving negative feedback of cortisol to the hypothalamic–pituitary–adrenal axis [5,89]. These results contrast with the finding from studies that compare cortisol levels in dairy cows between thermoneutral and hot periods when animals experience continuous stress. This is because the temperature in the hot chambers is kept stable for a predetermined period, whereas in the farm environment, animals can partially thermoregulate during the cooler night period. Additionally, in most modern farms, cooling systems reduce the environmental thermal load. In a recent study conducted on a Greek dairy farm, we found that in pregnant cows, cortisol concentrations were significantly higher during the summer than in the cooler periods of the year, despite the intense electronically operating cooling system that was applied in the summer [114]. It is known that there are significant differences in thermotolerance among various cattle breeds. For example, Zebu cattle are much more heat resilient compared to European breed cattle [123], as they have evolved to have reduced metabolic heat production and increased heat dissipation to the environment [124]. There are individuals with intrinsically increased thermotolerance exist within the European cattle breed populations. As stated above, we have examined the characteristics of phenotypically thermotolerant Holstein cows. These animals had the same milk yield as the thermosensitive animals, but under heat stress, they exhibited lower cortisol concentrations, and the preovulatory GnRH-induced LH surge was higher compared to the thermosensitive animals [125].

It is known that stress (either as physical or inflammatory) acts as gonadotropin secretion inhibitor, disturbing the normal ovarian cyclicity [126,127,128,129]. However, the exact mechanism by which stress disrupts normal gonadotrophin secretion is not fully understood. By administering low cortisol doses (lower than those measured during the typical stress response) to intact ewes, Breen et al. [130] confirmed their hypothesis that cortisol during the follicular phase suppresses the frequency of LH pulses, which interferes with the expression of the preovulatory estradiol rise and LH and FSH surges. This provides evidence that cortisol acts in the hypothalamus, suppressing the GnRH pulsatility. In addition, glucocorticoid receptors are present on granulosa cells, and it has been demonstrated that in the presence of glucocorticoids, the granulosa cells lose part of their response ability to gonadotrophic stimuli, leading to impaired steroidogenetic capacity [131,132]. Based on this, it can be postulated that the observed depressed LH and estradiol concentrations during the hot periods of the year, as reported by many researchers, can be explained by the prevailing high cortisol levels.

### 3.7. Prolactin

Prolactin is an anterior pituitary gland polypeptide hormone that is secreted from specialized cells known as lactotrophs or mammotrophs. Although initially recognized as lactation promoter [133], it is now clear that the hormone is involved in more than 300 biological functions. In addition, it has been observed that prolactin not exclusively synthesized in the pituitary, but other tissues such as the hypothalamus, placenta, uterus, and the spleen are also capable of synthesizing the hormone [134].

There is a consensus that HS induces over-secretion of prolactin, and it has been demonstrated that increases in prolactin secretion are positively associated with the elevation of the HS gradient [89,135]. For example, Tucker and Wetteman [136] reported a four-fold increase in prolactin concentrations when the ambient temperature increased from 21 °C to 32 °C. Do Amaral et al. [137] found significantly higher prolactin levels in heat-stressed dry dairy cows compared to non-stressed cows, while Igono et al. [138] reported significantly lower prolactin levels in cows kept under intense cooling measures such as shade provision, ventilation, and spraying, compared to cows without cooling. Other studies have reported that heat-stressed cows during the dry period have decreased estrogen and increased prolactin concentrations compared to cows in thermoneutral or cool conditions [139,140]. Ouellet et al. [141] showed that HS during the dry period, relative to thermoneutral conditions, alters the expression of prolactin and estrogen receptors in cows’ udders, suggesting that HS during the dry period changes the responsiveness of the mammary cells to estrogens and prolactin. The exact reason why HS modulates prolactin and estrogens remains unknown. It has been postulated that high prolactin levels may facilitate HSP synthesis and promote sweating and hair molting, which synergistically improve heat abatement [142].

There is some evidence to suggest a paracrine interaction between gonadotrophs and lactotrophs in the pituitary gland. Specific interactions between these cell types have been reported in immunocytochemical studies in various species [143,144], and the presence of prolactin receptor mRNA and protein have been shown to localize in gonadotroph cells of sheep [143].

A clear suppression of LH secretion by prolactin has been previously reported. In vivo, hyperprolactinemia has been shown to cause a significant reduction of LH release [145,146], leading to gonadal inactivity [147]. In rats, experimentally induced or lactational hyperprolactinemia inhibits gonadotrophin secretion and reduces LH response to GnRH administration [148,149]. In vitro studies in rats have shown that prolactin downregulates the HPO axis by acting mainly at the hypophysial level [150]. It has also been shown that induced hyperprolactinemia in sheep, through the administration of thyrotropin-releasing hormone, disrupts the estradiol-induced LH surge [151]. In an in vitro experiment using ovine pituitary cells collected during the anestrous and breeding season and cultured with prolactin and bromocriptine (a semisynthetic alkaloid that stimulates pituitary dopamine D2 receptors blocking prolactin synthesis and release) [144], it was revealed that that prolactin alone has a rather neutral effect on GnRH-induced LH synthesis, but the interaction between prolactin and dopamine is essential for the regulation of LH secretion.

The mode of action on prolactin on pituitary gonadotrophs or at the hypothalamic level remains elusive, but it is interesting to consider that in sheep, the seasonal anestrous is characterized by minimal gonadotrophin secretion and high prolactin concentrations [152]. Hence, it is logical to assume that HS-induced hyperprolactinemia in dairy cows plays a crucial role in summer subfertility, causing modifications in the pattern of LH secretion.

### 3.8. Follicular Development

In cattle, follicular development, during the estrous cycle, there occurs in a wave-like pattern consisting of two, three, and rarely four follicular waves [153,154,155]. In this process, only antral follicles participate. However, the required time for primordial (non-antral follicle) to reach the ovulatory stage is approximately 180 days, while that for an early antral follicle is 40 to 50 days. In other words, their development duration is two-fold longer than the duration of the estrous cycle [156]. In areas affected by the summer HS, a common and undeniable observation is that fertility is restored well after the last exposure of cows to high ambient temperatures, and this rarely occurs before the mid-autumn [157]. This observation can be explained either as the result of direct negative effects imposed by HS on the pool of small, even preantral follicles, as a strong carryover effect of HS on the oviductal environment, or the combination of both conditions. Nonetheless, the long-term deterioration of the oocyte quality after exposure to HS is well documented [158]. Follicular development includes the gonadotrophin-independent phase of primordial and primary follicles, which is characterized by excessive mitoses of granulosa cells and synthesis of various factors involved in follicle formation, such as various cytokines, growth factors, anti-mullerian hormone, and gap-junction components. As early as the primordial stage, FSH receptors are present on granulosa cells, but these follicles can sustain their development in the absence of FSH support [159,160].

Although there is no conclusive in vivo evidence for the direct effect of HS on pre-antral follicles, when pre-antral follicles were individually cultured in vitro for seven days and exposed to 41 °C for 8 h daily, they had decreased viability and developmental potential compared to non-stressed controls. This decrease is likely attributed to increased oxidative stress, as revealed by the overexpression of *SOD1* mRNA in heat-stressed follicles [161]. Preantral follicles (primordial and primary), exposed for a single 12-h time window to elevated temperature (41 °C) during a 7-day in vitro culture, showed increased ROS production. Interestingly, HS facilitates the activation of the primordial follicles, which is likely attributed to enhanced and accelerated cellular metabolism [162]. It is well-documented that ROS act as second messenger at certain concentrations, but at higher levels, they cause cell damage that is detrimental to follicular development [163,164]. In a more recent in vitro study, it has been shown that the ovarian pool of primordial follicles is highly affected by HS. After exposure of ovarian cortical tissue to 41 °C for only 2 h, the number and the viability of granulosa cells were significantly decreased, mainly due to increased apoptosis [165]. These finding support the notion that the follicular reserve of preantral and early antral follicles are sensitive to HS, and this condition undermines the reproductive performance of cows beyond the period of HS exposure.

In an in vivo study involving controlled exposure of dairy cows to HS and successive induction of follicular development through selective ultrasound-guided dominant follicle aspiration every eight days, it was reported that HS increases the number of developing small (2 to 5 mm) follicles. This increase was attributed to a possible induction of metabolites and/or growth factors by the acute HS [166]. However, ultrasound-guided aspiration of the pool of medium-sized follicles previously affected by HS led to the emergence of healthy follicles containing oocytes with increased developmental competence [158].

Early observations by Ryan and Boland [167] demonstrated increased incidence of double ovulations during the summer, which is indicative of a disruption in normal follicular development under prolonged exposure to high environmental temperatures. The widespread application of transrectal ultrasonography in bovine practice has allowed for monitoring of follicular development in real time. Since the early 1990s, several studies have provided compelling evidence that HS interferes with normal follicular development and turnover in dairy cows [70,72,113,168]. There is a general consensus that HS alters the pattern of follicular dynamics, the structural characteristics of the dominant follicle, and the dominant follicle turnover in cattle. The destructive effects of HS on dairy cows have been extensively studied and reviewed in many comprehensive papers. The main detrimental effects of HS on follicular development and function can be summarized as follows: (a) attenuated dominance, which results in the growth of large subordinate follicles, (b) reduced size of the dominant follicle, (c) impaired growth of medium-sized follicles, and (d) early emergence and prolonged dominance of the pre-ovulatory follicle, which compromises oocyte quality, leading to reduced fertilizing capacity of the oocyte and compromised embryo development potential [70,72,169,170].

## 4. Cumulus Complexes, Oocyte Complexes, and the Early Embryo

The effects of HS on the overall fertility of dairy cows, including disruptions in estrus expression, low conception rates, and increased embryonic mortality, have been extensively documented. However, the relative contribution of different component factors, such as oocyte quality, fertilizing capacity, gamete transport, early embryo quality, developmental competence, and the oviductal environment, can be only retrospectively evaluated, and they are extremely difficult to accurately assess.

In vitro studies provide reliable and reproducible tools to study the effects of HS on oocyte and early embryo development, although the in vitro conditions can never perfectly replicate naturally occurring conditions. Despite this limitation, in vitro embryo production methodologies provide a dynamic model for the continuous study of oocytes, cumulus cells, and early embryos under well-defined environments. The following sections present data derived from findings from in vitro embryo production studies.

### 4.1. The Oocyte and the Surrounding Cumulus Cells (COCs)

The in vitro maturing oocyte acquires developmental competence during the first 12 h of in vitro maturation. During this time period, enhanced de novo protein synthesis occurs, and the oocyte transitions from the inductive to the synthetic phase [171,172]. Maturation Promoting Factor (MPF) contributes to the activation of MAPK and ERK1/2 to promote phosphorylation of proteins involved in nuclear membrane formation, chromatin condensation, and microtubule reorganization [173,174].

High temperatures cause functional and structural defects in the maturing oocyte, including a failure to progress to Metaphase II [51,175], early arrest in metaphase II (accelerated maturation) [52], incomplete migration of cortical granules, impaired mitochondrial distribution, and increased ROS accumulation [51,175]. Increased oxidation triggers apoptotic phenomena associated with increase nuclear DNA fragmentation [26], impaired distribution and function ofmitochondrial and cortical granules [175,176], and disrupted microfilament and microtubule rearrangement [51,177]. Therefore, the in vitro maturing oocyte is highly sensitive to HS, especially during the period around the second meiotic arrest of metaphase II [6,175]. According to the available bibliographic data, the exposure of immature oocytes to 41 °C for 6 or 12 h either affects [51,53], or does not affect [52,178] their fertilizing capacity and the ability of the fertilized ovum to proceed to the first mitotic divisions. Nevertheless, there is an agreement that the further developmental competence of these oocytes is seriously affected, as blastocyst formation rates from heat stressed oocytes are significantly suppressed. After exposure of COCs to increased temperature during in vitro maturation, a reduced embryo yield has been reported in many studies [18,54,179,180,181,182]. In most of these studies, the heat exposure lasted for 12 to 24 h. We have shown that only six hours exposure (from the second to the eighth hour of IVM) of in vitro matured COCs to 41 °C is sufficient to cause significant reduction in cleavage and blastocyst formation rates [54].

**Figure 2:**
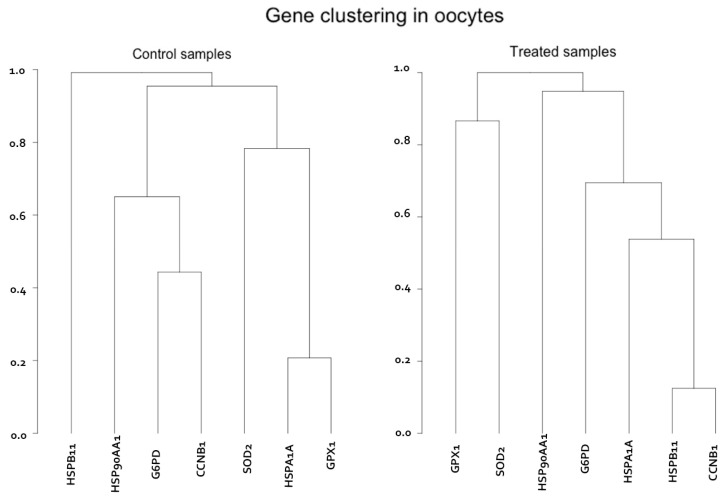
Hierarchical gene clustering in oocytes matured in vitro at 39 °C (control) and at 41 °C for 24 h from the 2nd to 8th hour, and at 39 °C thereafter (treated) (figure from Stamperna et al., 2020) [54].

Although short-term temperature elevation during IVM does not affect HSP gene expression, the HS conditions affect the orchestrated gene expression, as revealed by the correlation coefficients, leading to a tight clustering of HSP expression with cell cycle regulatory genes (*HSPA1A*, *HSPB11*, and *CCNB1*). This highlights a common response to elevated temperature by key players in the cell cycle and heat tolerance (Figure 2) [54]. Furthermore, there is a strong tendency for upregulation of antioxidant genes under HS conditions (*GPX1*, *MnSOD*, *G6PD*), which are crucial components of the antioxidant mechanisms of the cell and have been associated also with oocyte competence [183,184,185].

On the other hand, the short-term temperature elevation is associated with significant changes in the expression of specific genes in cumulus cells, namely *HSP90AA1*, *G6PD*, and *CPT1B* [54]. This suggests that oocytes acquire antioxidant protection through cumulus cells. *HSP90AA1*, which encodes for a cytosolic molecular chaperone, ensures the proper folding of misfolded proteins. It also interacts with the phospholipid bilayers of the membrane, enhancing its stability [186]. Elevated levels of *HSP90AA1* were also observed in heat-stressed embryos after a short-term exposure to heat stress [63]. It appears that when cumulus cells are cultured under high temperatures, a cellular metabolic adaptation occurs towards lipid exploitation and lower glucose metabolism. This is manifested by the downregulation of *G6PD* and the up regulation of *CPT1B*, along with coordinated expression patterns between these genes. Furthermore, genes related to glucose metabolism (*LDHA*, *G6PD*, and *SLC2A1*) are tightly correlated under heat stress conditions [54].

Oocytes respond to external thermal stimuli by increasing the production of HSPs, which act protectively by modulating protein synthesis and providing antiapoptotic functions [187,188].

**Figure 3:**
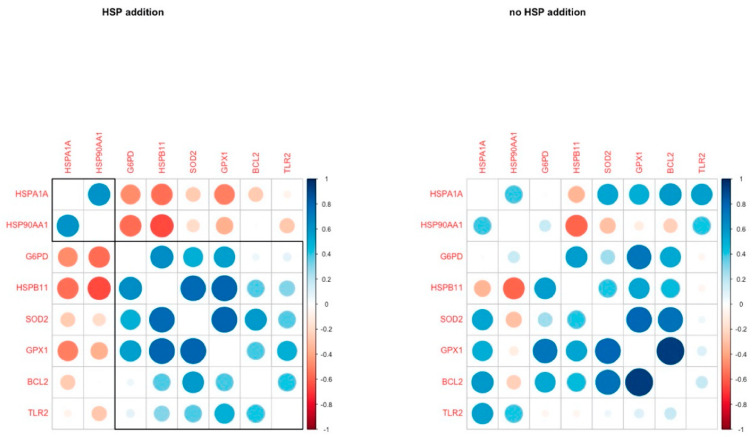
Pairwise correlation coefficients of genes under study in oocytes with (**left**) and without (**right**) HSP70 addition to the culture medium (figure from Stamperna et al., 2021) [125].

The addition of HSP70 at a dose of 5 ng/mL to the in vitro maturation medium of heat stressed COCs, which is the average concentration found in heat-stressed cows [31], does not affect the cleavage rate. However, it attenuates the negative effects of HS on oocyte maturation and restores the yield of blastocysts to levels close to those of oocytes matured at thermoneutral conditions [118]. The external addition of HSP70 reduces the expression of the endogenous HSPs, both in control and heat stress conditions, while increasing the expression of antioxidants as well as anti-apoptotic genes (*SOD2*, *GPX1*, *BCL2*) in oocytes [181]. These changes contribute to cellular protection against the stressors and are associated with improved oocyte viability and competence. The addition of HSP70 caused an upregulation of the antiapoptotic gene *BCL2* and the antioxidant genes *SOD2* and *GPX1* in oocytes. In the resulting blastocysts, it altered the expression of the anti-toxic *AKR1B1*, the anti-apoptotic *GPX1*, *HSPA1A*, and *BAX*, and genes that regulate the stress response and contribute to embryo growth such as *ATP1A1*, *IGF1*, and *TLR2* [189]. Furthermore, the addition of HSP70 dramatically altered the patterns of gene expression, leading to more coordinated patterns, particularly between HSPs and antioxidants genes (*HSPB11*, *SOD2*, *GPX1*, *HSP90AA1*, and *HSPA1A*) (Figure 3). Therefore, it can be postulated that while HSP70 can provide a strong thermal protection to COCs, the COCs themselves are not capable of self-protection under harsh environmental stress.

It is noteworthy that embryos produced from heat-stressed oocytes exhibited particular characteristics. For example, compared to untreated controls, these embryos presented lower *DNMT1* and higher *PLAC8* expression. This indicates reduced potential for epigenetic modifications and an increased likelihood of developing a well-functioning placenta, respectively [18], which are related to increased quality or robustness. The latter findings are partly confirmed by the results from an in vivo study according to which, between days 33 to 36 of pregnancy, the placenta derived Pregnancy Associated Glucoproteins (PAG) levels are significantly higher during the summer months, when cows are continuously exposed to HS, than during the cooler months of the year [20].

### 4.2. Early Embryos

As stated above, the early embryo is extremely vulnerable to high temperatures and exhibits biphasic thermosensitivity. It is highly thermosensitive until the early morula stage, after which thermotolerance gradually increases [49,50,51]. At early stages of embryonic development, increased transcription activity and protein synthesis is observed at the time when the embryonic genome becomes activated [60,61]. At this stage, several HSPs [62] and antioxidants enzymes [63] are overexpressed, strengthening the molecular defense mechanisms of thermotolerance of the developing embryo. Many in vivo and in vitro studies have shown that exposing oocytes and early embryos to HS does not prevent the embryo from developing into the blastocyst stage. However, these blastocysts carry the defects that were imposed by HS during preceding stages of oocyte and/or embryonic development. This can partially explain the low conception rates and increased embryo losses during the summer. Aberrant expression of many genes has been detected in blastocysts originating from heat-stressed COCs, and the set of genes presenting altered expression in oocytes and cumulus cells differs from those in blastocysts [54]. Nevertheless, it has been documented that transcriptional activity for HSPs and HSP70 synthesis is detectable even before embryonic genome activation [64,65]. It has been also shown that early embryos exhibit a rapid reaction to short-term moderate temperature increases, which is evidenced by the steadily increasing expression of mRNA for *HSPA1A* and *HSP90AA1* after exposure of early morulae to a 1.5 °C temperature elevation (40.0 °C instead of 38.5 °C) for 2 to 8 h [63]. In a recent study, we exposed early morulae to 41 °C for 24 h (48th to 72nd hour of in vitro culture) in the presence or the absence of HSP70. The HSP70-primed embryos developed into blastocysts in a significantly higher proportion compared to the non-primed controls. However, they still they lagged behind compared to the non-stressed controls [181]. In conjunction with the findings on the effect of HS on the maturing oocyte, these findings are indicative of two phenomena: first, the endogenous synthesis of HSPs is not sufficient for the early embryo to withstand thermal stress, and second, interventions during oocyte maturation stages are more efficient and appear to persist until the blastocysts stage. These effects may be attributed to transgenerational inherited epigenetics changes.

Despite achieving thermotolerance, the embryos remain sensitive to thermal insults. This sensitivity is evident in the increased occurrence of late embryonic losses during the summer, which could reach up to 40% of confirmed conceptions [190]. We have shown that the embryo losses that between days 24 and 32 are three times higher during the summer (18.8%) compared to the winter (5.6%). In the same study, it was revealed that, compared to the winter, the trophoblastic PAG levels on day 36 were significantly higher during the summer, despite the fact that the embryos survived and developed under significantly lower concentrations of progesterone [20]. This finding provides an in vivo justification for the increased PLAC8 expression in blastocysts derived from heat-stressed oocytes [54].

## 5. Oviduct—Gravid Uterus

The oviduct plays a significant role in mammalian reproduction, as it is involved in gamete transport, fertilization, mother–embryo crosstalk, and migration of the embryo into the uterus. The oviduct accomplishes most of its roles through the oviductal fluid, which contains carbohydrates, proteins, salts, metabolite lipids, and extracellular vesicles (EVs) [191]. The composition of the oviductal fluid is dynamic and affected by cyclic changes in steroid hormone concentrations throughout the estrous cycle, and by the presence or absence of the gametes and the early embryo [192]. It is noteworthy that the presence of the embryo mainly alters the expression of immune-related genes, and their downregulation is essential for the survival of the semi-allogeneic embryo [192,193]. Studies have shown that the oviductal fluid undergoes specific changes as a response to heat stress, where the secretion of PGE2 and HSP90AA1 is upregulated in specific regions of the oviduct; however, the secretion of PGF2a is not affected. This imbalance is thought to contribute to impairments in gamete transport, resulting in reduced fertility during heat stress [194].

The mother–embryo crosstalk begins at the oviductal environment and is essential for proper development of the embryo, as it enhances the embryo’s heat stress resistance, its gene expression, and its developmental potential (reviewed in Kolle et al. [195]). This crosstalk is primarily mediated by the epithelial cells of the oviduct (bovine oviductal epithelial cells, BOECs) and their secretions, which include amino acids, pyruvate, antioxidant enzymes, and embryo trophic factors (OVGP1) [196]. OVGP1 is an essential factor that interacts with the zona pellucida (ZP), resulting in the formation of the barrier against polyspermy. In an in vitro experiment conducted by Rapala et al. [197], the effect BOECs in the culture medium of developing embryos under heat stress conditions was investigated. They showed that HS did not affect the viability of BOECs; however, the expression of HSP70 was upregulated, while the expression of OVGP1 was significantly downregulated, negatively affecting the developmental potential of the embryos.

In a recent study [19], a comparative transcriptomic analysis was conducted using RNASeq of BOECs during a thermoneutral period (spring) and a HS period (summer). The results revealed that the main pathways of the expressed genes were associated with protein synthesis, mitochondrial biogenesis, and the adaptive immune system, which aligns with the functions of BOECs. Differential gene expression between seasons revealed several non-coding RNAs with differential expression patterns. Long, non-coding RNAs were found to exhibit differential expression under HS in many previous studies involving dairy cows [198,199], and they are consequently considered to be important components of the heat shock response in mammals [200] and regulators of the immune response [201]. In the same study [19], several immune-related genes were found to exhibit differential expression. This result, along with the significant changes observed in progesterone levels between periods, is indicative of the detrimental effects of over-activation of the immune system during HS on fertility potential. Among other genes with significant changes in gene expression, those of haptoglobin, myosin light chain kinase, and antileukoproteinase were highlighted. Haptoglobin, known for its role in regulating gametes interaction and embryo development [202], as well as its immunosuppressive function, was downregulated during heat stress, negatively affecting reproductive potential. Myosin light chain kinase regulates the contractile activity of the oviduct by phosphorylating myosin regulatory light chains, and its overexpression may be associated with impaired contraction and relaxation of the oviduct during heat stress. This impairment is also affected by the imbalance of prostaglandins secretion [194]. Antileukoproteinase acts as an immunosuppressor by inhibiting proinflammatory cytokines, and it mediates the gametes interactions and protects the acrosome reaction. Its downregulation during heat stress may have detrimental effects on immune system activation as well as in the “safeguarding” of the pregnancy.

In recent years, there has been a growing interest in decoding the role of the oviductal extracellular vesicles in embryo development and maternal–embryo communication. Extracellular vesicles (EVs) are membrane-enclosed nanoparticles that are detected in the extracellular space, containing proteins, lipids, mRNAs, and miRNAs cargo [203,204]. Even at prehatching stages, the early embryo is able to uptake oviductal EVs, indicating that EVs can migrate from the oviductal fluid into the embryo by crossing the intact zona pellucida, thereby playing an additional embryotrophic role [205]. Zygotes co-cultured in vitro in the presence of oviductal EVs develop into blastocysts of improved quality, which is validated by the expression of the transcript abundance of genes related to metabolism, water channel traffic, and epigenetics, as well as improved cryotolerance compared to control embryos [206]. We have recently shown that the concentration of EVs in the ipsilateral oviduct to the CL at the early stages of the luteal phase (days 3 to 4) is reduced during the hot summer months compared to thermoneutral periods. This impaired embryotrophic condition can be considered as an additional causative factor for summer subfertility in dairy cows [19].

## 6. Conclusions and Considerations

It is undeniable that HS is undermining cow productivity and fertility, directly jeopardizing the viability and sustainability of the global dairy industry. The mechanisms through which HS disrupts the endocrine, reproductive, immune systems, and metabolism of cows are only partially understood due to their complexity and multiple interactions. Advanced nutritional interventions aiming to increase dry matter intake and improve feed utilization, as well as hormonal treatments targeting improved conceptions per insemination, somewhat mitigate the situation but fall short of solving the problem. While widely used techniques and technologies for cooling the cows and regulating the microclimate of the farms address the problem of low summer productivity, they have negligible effect on subfertility. Additionally, their high installation and operational costs, as well as their significant environmental footprint, indicate that their effectiveness must be meticulously re-evaluated. It appears that a solution to the problem should be pursued through the combination of science and technology. The vision of creating a new cattle population with inherent increased thermal resilience, requiring only moderate artificial cooling, is not unrealistic. This population could be developed through intense selection for thermotolerance [38,207] and productivity using the available modern omics technologies (genomics, transcriptomics, proteomics, metabolomics, etc.). Carefully selected individuals could be subsequently bred through planned breeding programs, predominantly using embryo transfer technologies. However, as indicated by several genetics and genomics studies discussed in Section 2, the polygenic nature of thermotolerance, the distinctive mechanisms involved in HS response, and the low heritability estimates of these traits make this effort challenging. Nonetheless, the available technologies can be integrated into this effort to improve the genomic thermotolerance of the herds.

## Data Availability

Not applicable.
